# Complete Genome Sequences of Three Border Disease Virus Strains of the Same Subgenotype, BDSwiss, Isolated from Sheep, Cattle, and Pigs in Switzerland

**DOI:** 10.1128/genomeA.01238-17

**Published:** 2017-11-09

**Authors:** Hanspeter Stalder, Sabrina Marti, Franziska Flückiger, Nathalie Renevey, Martin A. Hofmann, Matthias Schweizer

**Affiliations:** Institute of Virology and Immunology, Bern and Mittelhäusern, Switzerland, and Department of Infectious Diseases and Pathobiology, Vetsuisse Faculty, University of Bern, Bern, Switzerland

## Abstract

We report here the complete genome sequences of three border disease virus (BDV) strains of the same subgenotype isolated in Switzerland from a sheep, a cow, and a pig, respectively. This is the first report of full-length sequences of a tentatively new subgenotype isolated from three different species of cloven-hoofed farm animals.

## GENOME ANNOUNCEMENT

Border disease virus (BDV) belongs to the genus *Pestivirus*, which includes important animal pathogens, such as bovine viral diarrhea virus (BVDV) and classical swine fever virus (CSFV). BDV occurs worldwide (1, 2), and several subgenotypes have been described to date (3, 4). BDV infections occur mainly in sheep and goats but also in cattle, pigs, and wild even-toed ungulates (2). We describe here the full-length sequences of three BDV strains isolated in Switzerland from a sheep, a cow, and a pig. They form a new, yet unclassified, BDV subgenotype. The first isolates of this type were isolated in 2006 in Switzerland and provisionally named BD Switzerland (5) or BDSwiss (6–8) or remained unclassified (4, 9, 10), and partial sequences (5′ untranslated region [UTR], N^pro^) were deposited in GenBank (accession no. JQ994199, JQ994200 GU244490, and GU244489). Recently, BD viruses of this subgenotype were detected in goats and chamois in Italy and were tentatively labeled BDV-8 (11, 12).

Samples R4785/06 (also named CH-BD4), R9336/11, and BD35-15 were obtained from a female white alpine sheep in 2006, a crossbreed (Braunvieh × Limousin) male cow in 2011 (7), and a female domestic pig in a zoo, respectively. Viruses from blood of sheep and cattle were isolated on bovine turbinate cells, whereas SK6 cells were used for porcine blood. RNA was isolated using the QIAamp viral RNA minikit (Qiagen AG, Hombrechtikon, Switzerland), and reverse transcription-PCR (RT-PCR) (Qiagen OneStep RT-PCR kit) was performed according to the manufacturer’s instructions. PCR fragments were purified with the QIAquick PCR purification kit (Qiagen). DNA-Sanger cycle sequencing with BigDye Terminator chemistry (version 3.1) and capillary electrophoresis (ABI 3730xl DNA analyzer; Applied Biosystems) were performed at Microsynth (Balgach, Switzerland). The 3′ ends were determined by a simplified protocol for rapid amplification of cDNA ends (RACE) (13) with direct sequencing of the amplification products or by cloning into pCRTM 4-TOPO (Invitrogen). The 5′ ends were determined by a 5′ RACE kit (Invitrogen or Roche). The electropherograms were assembled with SeqMan II version 5.01 (DNAStar, Inc., Madison, WI), and sequences were analyzed with Clone Manager 9 professional edition (Scientific & Educational Software, Cary, NC) and the MEGA program, version 6 (14).

The complete genomes of the isolates R4785/06, R9336/11, and BD35-15 comprise 12,318, 12,311, and 12,309 nucleotides (nt), with 5′ UTRs of 369, 377, and 375 nt and 3′ UTRs of 261, 246, and 246 nt, respectively. All polyprotein-coding sequences are 11,685 nt long and code for 3,895 amino acids (aa). The bovine and porcine isolates are more similar to each other (99% nt, 99% aa) than to the ovine strain (86% nt, 92% aa). In the N^pro^ genomic region, the sequences of the strains reported recently from Italy (11, 12) match 82 to 92% (nt) and 86 to 93% (aa) to the corresponding regions in our strains.

This is the first report of complete consensus sequences of three strains of the same BDV subgenotype, BDSwiss (later also named BDV-8), obtained from a sheep, a cow, and a pig in Switzerland, which will further assist investigations on the epidemiology and evolution of pestiviruses in different host species.

### Accession number(s).

The complete sequences of the isolates R4785/06, R9336/11, and BD35-15 have been deposited in GenBank under the accession no. MF102260, MF102261, and MF102262, respectively.
